# Asymptomatic individuals can increase the final epidemic size under adaptive human behavior

**DOI:** 10.1038/s41598-021-98999-2

**Published:** 2021-10-05

**Authors:** Baltazar Espinoza, Madhav Marathe, Samarth Swarup, Mugdha Thakur

**Affiliations:** Biocomplexity Institute and Initiative, Network Systems Science and Advanced Computing Division, University of Virginia, Charlottesville, VA USA

**Keywords:** Infectious diseases, Human behaviour, Applied mathematics

## Abstract

Infections produced by non-symptomatic (pre-symptomatic and asymptomatic) individuals have been identified as major drivers of COVID-19 transmission. Non-symptomatic individuals, unaware of the infection risk they pose to others, may perceive themselves—and be perceived by others—as not presenting a risk of infection. Yet, many epidemiological models currently in use do not include a behavioral component, and do not address the potential consequences of risk misperception. To study the impact of behavioral adaptations to the perceived infection risk, we use a mathematical model that incorporates the behavioral decisions of individuals, based on a projection of the system’s future state over a finite planning horizon. We found that individuals’ risk misperception in the presence of non-symptomatic individuals may increase or reduce the final epidemic size. Moreover, under behavioral response the impact of non-symptomatic infections is modulated by symptomatic individuals’ behavior. Finally, we found that there is an optimal planning horizon that minimizes the final epidemic size.

## Introduction

Non-symptomatic (pre-symptomatic and asymptomatic) individuals have the potential to affect the course of an epidemic through silent infections. Transmission events in the absence of symptoms have been documented for different diseases, including the ongoing COVID-19 pandemic^1–3^. The emergence of the SARS-CoV-2 virus challenged the scientific community to promptly uncover its pathogenesis and transmission dynamics in order to fight infections and achieve disease containment. The potential transmission of COVID-19 during the pre-symptomatic and asymptomatic stages was recognized relatively quickly^4,5^. Containment efforts involving contact tracing and testing have identified non-symptomatic individuals as major drivers of COVID-19 transmission in a number of countries^3,6–10^. However, the impossibility of identifying non-symptomatic individuals without testing poses a major challenge for disease containment. Yet most countries only test symptomatic individuals^11^. Ideally, large scale random testing with appropriate test sensitivity is required to characterize the progression routes of infection^12,13^. Without random testing, the role of silent infections is hard to identify. The problem is made more difficult by the fact that the asymptomatic/symptomatic ratio, as well as the infectiousness potential of COVID-19 asymptomatic individuals is uncertain^14–16^. Studies estimating asymptomatic relative infectiousness report highly variable results, ranging from (0–40%)^7,10,17^ to (40–70%)^11,12,18–20^.

Absent testing, infections produced by non-symptomatic individuals are difficult to prevent and to track, due to the lack of apparent illness and to the fact that non-symptomatic individuals are unaware of the infection risk they pose to others. Non-symptomatic individuals may perceive themselves—and being perceived by others—as not representing an infection risk, potentially starting infection chains that are not detectable through contact tracing^9^. Yet many epidemiological models currently in use do not include a behavioral component, and do not address the potential consequences of risk misperception.

To get a measure of the risks posed by infectious non-symptomatic individuals we consider behavioral responses to perceived risk of infection. Behavioral responses aimed at mitigating disease risk include social distancing by both susceptible and infected individuals, increased use of protective equipment, and better hygiene. Collectively, the suite of behavioral responses taken by individuals have been characterized as a *behavioral immune system* at the population level^21^. Modern mathematical models envision epidemics as complex systems in which behavioral responses, at different scales, both drive and are driven by the disease transmission process. A number of different mathematical modeling frameworks have been used to understand interactions between disease dynamics and behavioral responses^22–24^.

Before the vaccines against COVID-19 infections were widely available, behavioral changes mainly took the form of variation in contact rates over the epidemic period. To study individuals’ management of decentralized social distancing in this paper we use per-capita contact rates as the mechanism by which disease is transmitted and benefits are obtained. In other words, we assume economic productivity depends on social interactions^25,26^. We apply the framework by Fenichel et al. in^27–29^, to study how the behavior of infectious exposed and asymptomatic individuals affects the spread of a disease. Specifically, we use the impact of behavioral adaptations to disease risk to understand the effect of silent infections on the transmission dynamics and on the final epidemic size. As in the work by Fenichel et. al., we use a set of ordinary differential equations to model disease progression, and a decentralized Markov decision framework to model the strategic behavior of individuals in different health classes. Behavioral changes are modeled as adjustments in the contact decisions made by individuals seeking to maximize the net benefits offered by contacts with others, where contacts also carry a risk of infection. Specifically, we model the response to disease risk as a trade-off between benefits secured through contact with others, and costs associated with the probability of infection due to contact with others expected to occur over some finite planning horizon. That is, individuals chose their daily contact rates, given their understanding of infection risks, so as to maximize the discounted flow of net benefits over a given planning horizon. The decision process accounts for expectations of future utility, future risk of infection and potential future transitions to alternative health states, based on a projection of the future system’s state over a finite planning horizon. The forward looking decision making process in the modeling framework we use is a critical modeling component. A variety of models use the current and past system’s states to derive behavioral decisions^30–32^. In the proposed framework individuals decisions not only depends on the current system’s state but on a projection of the future. This allow us to explore the impact of planning over short- and long-term periods on the decision process over the epidemic period.

Understanding of infection risk is assumed to be determined by vulnerability cues^21^. Since most social interactions require immediate evaluation of the infection risk, individuals are biased towards easily observable cues—specifically the presence or the absence of symptoms. It follows that there will be at best a weak behavioral response to individuals exhibiting mild or no symptoms. Specifically, we suppose that the impact of non-symptomatic (exposed and asymptomatic) individuals on the transmission dynamics depends on two misperceptions: (i) non-symptomatic individuals are treated as not infectious; (ii) uninfected and non-symptomatic infectious individuals behave as if they are susceptible.

Taking variation in the final epidemic size as a measure of the impact of asymptomatic infections, we consider the net effect of these two misperceptions on the behavior of the non-symptomatic population. The risk-avoiding behavior of non-symptomatic but infectious individuals who perceive themselves to be susceptible, is balanced against the risk-increasing behavior of susceptible individuals who perceive non-symptomatic individuals as non-infectious. Moreover, the impact of the non-symptomatic population’s behavior is conditioned by the behavior of the symptomatic but still socially active population. In the US, as in many other countries, non-pharmaceutical pro-social precautionary measures by infected but socially active individuals are recommended but not mandatory^33,34^. Consequently, it is expected that only a fraction of the infected but socially active population will comply with health authority recommendations. We test the strength of the impact of variations in the behavior of the non-symptomatic population to variations in the proportion of the symptomatic population adopting pro-social behaviors.

We find that under behavioral adaptation an epidemic driven by both symptomatic and asymptomatic cases may produce a greater final epidemic size than the analogous epidemic solely driven by symptomatic cases. Individuals’ risk misperception, by playing a dual role on the behavioral response produced during an epidemic, may ameliorate or exacerbate the epidemic. By contrast, constant contacts models, may find the final epidemic size monotonically decreases when a proportion of infections result in asymptomatic cases. A related result using an epidemiological model including behavioral response via a game theoretic approach was recently derived by Hota et al.^35^. The authors found that there is a trade off between the disease prevalence and the individuals activity rates that impacts disease persistence.

Moreover, under behavioral response the impact of silent infections is modulated by the symptomatic individuals’ behavior. The lower the symptomatic individuals’ contact rate, the greater the impact of silent infections on the attack rate, the proportion of the population infected over the epidemic. Finally, we found that there is an optimal planning horizon that minimizes the final epidemic size regardless of the proportion of asymptomatic cases and their relative infectiousness.

## Results

Since the model is not amenable to an analytic solution, we numerically explore the implications of adaptive behavior and risk misperception on the epidemic dynamics and on the attack rate. We assume per-contact utility to be independent of health status and use the single peaked utility function $u_t=\left( b C_{t}^{h}-(C_{t}^h)^2\right) ^{\nu }$, where $C_{t}^{h}$ represents the contact rate of a typical individual with health status *h* at time *t*, and where the maximum number of contacts available per time (*b*) and the utility function shape parameter ($\nu$), are fixed over time. Therefore, $u(h,C_t^h)$ represents the immediate utility a typical individual in health class *h* obtains by making *C* contacts at time *t*.

Since preferences are single-peaked each individual has a unique most preferred contact rate and, although the utility function is symmetric around the optimal contact rate $C^*=b/2$, we restrict behavior adaptations to reductions in the contact rate. In Appendix C we explore the impact of changes in the utility function on the adaptive behavior produced.

In the absence of appropriate behavioral data, we assumed individuals make an average of $b=48$ contacts per day and that future utility is discounted at the rate of 5% per year ($\delta =0.99986$), and the utility function parameter value is assumed to be $\nu =0.1$^27,29^. We explore the impact of variations in these parameter values in Supplementary Appendix C. We calibrate the behavior model by letting the basic reproductive number of the constant contact rates model to be consistent with early disease dynamics of the COVID-19 pandemic. Exposed individuals are assumed to exhibit a 5 days latency period ($\kappa =1/5$) with a reduced infectiousness of $\rho =0.25$^4,36^. Infected individuals recover and cannot infect others on average after 9 days ($\gamma =1/9$) of symptoms onset^37^. For our baseline parameters we assume 50% ($\sigma =0.5$) of the infections become asymptomatic with relative infectiousness of $\epsilon =0.4$^10^, and all symptomatic individuals to be non-compliant ($l=1$). These baseline parameters with a per-contact likelihood of infection $\beta =0.01324$, generate a basic reproductive number of 2.4,^38,39^. The set of parameters used in our numerical experiments, unless otherwise indicated, are collected in Table 1.

**Table 1:** Constant contact rates and adaptive behavior model baseline parameters.

Parameter	Description	Baseline value	Reference
$\nu$	Utility function shape parameter	0.1	^27^
$\delta$	Discount factor	0.99986	^27^
*b*	Maximum number of contacts per day	48	^27^
$\beta$	Likelihood of infection	0.01324	^27,38^
$\kappa$	Latency rate	1/5	^4,36^
$\gamma$	Recovery rate	1/9	^37^
$\tau$	Planning horizon length	14	Assumed
$\rho$	Exposed ind. infectiousness	0.25	Assumed
*l*	Proportion of non-compliant ind.	1	Assumed
$\eta$	Compliant ind. relative infectiousness	0.4	^10^
$\epsilon$	Asymptomatic ind. relative infectiousness	0.4	^10^
$\sigma$	Proportion of asymptomatic ind.	0.5	^14,15^

We now use model (1) with varying contact rates to study the impact of susceptible and non-symptomatic individuals' behavior on the transmission dynamics and on the attack rate.

### Risk misperception has the potential to increase the attack rate

**Figure 1:**
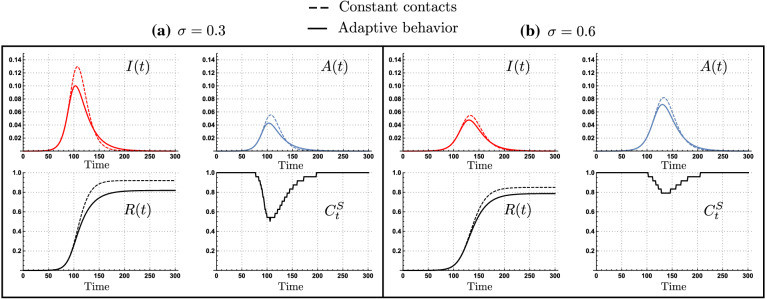
Disease dynamics under adaptive behavior (thick curves) and constant contact rates (dashed curves). The scenarios where 30% (**a**) and 60% (**b**) of cases become asymptomatic show differential behavioral response $(C^S_t)$ as a function of the risk perception, impacting the attack rate. Parameters $\tau =14,\nu =0.1, \epsilon =0.4,C^I=\max C_t$, $l=1$ and $\rho =0.25$.

Figure 1 shows selected simulations for disease dynamics under constant contact rates (dashed curves), and under adaptive behavior (solid curves). It reports two scenarios: in panel (a) 30% ($\sigma =0.3$) of all infections are asymptomatic, and in panel (b) 60% ($\sigma =0.6$) of all infections are asymptomatic. In panel (a), behavioral adaptation reduces the contact rates of susceptible and non-symptomatic individuals $(C^S_t)$ down to 50%, while in panel (b), there is a weaker behavioral response, reducing the susceptible and non-symptomatic contact rates to 80%.

While adaptive behavioral responses to disease risk reduce the contact rates in both cases, our numerical experiments show that the level of contacts reduction is sensitive to the proportion of infections that are asymptomatic. Specifically, the previous simulations show the impact of risk misperception to silent infections. In the scenario of $\sigma =0.3$, the epidemic is mainly driven by symptomatic transmission, in consequence the perceived risk associated to the symptomatic individuals prevalence produce a strong behavioral response. In counterpart, in the scenario of $\sigma =0.6$ the epidemic is mostly driven by asymptomatic transmissions. In this scenario, the perceived risk associated to the symptomatic individuals prevalence, produce a weaker behavioral response compared to the scenario of $\sigma =0.3$.

**Figure 2:**
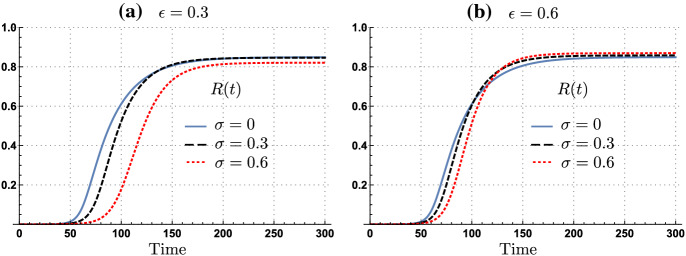
Time evolution of the proportion of recovered population for scenarios with 0%, 30%, and 60% of asymptomatic cases under adaptive behavior model. The impact of the asymptomatic subpopulation on the attack rate depends upon its relative infectiousness. (a) Shows that increments of the asymptomatic subpopulation having low infectiousness ($\epsilon =30\%$), decreases the attack rate. (b) Shows that increments on the asymptomatic cases having high infectiousness ($\epsilon =60\%$), increases the attack rate. Parameters $\tau =14,\nu =0.1, C^I=\max C_t$, $l=1$ and $\rho =0.25$.

We now explore the impact of the asymptomatic individuals’ relative infectiousness on the attack rate. Figure 2 shows that the impact of silent infections depends upon the relative infectiousness of asymptomatic individuals. Our simulations show that under low infectiousness of asymptomatic individuals ($\epsilon =30\%$), the attack rate decreases as the proportion of asymptomatic cases increases. However, if asymptomatic individuals are relatively highly infectious, ($\epsilon =60\%$), the attack rate increases as the proportion of asymptomatic cases increases.

Intuitively, the non-monotonic dynamics of the attack rate for the adaptive behavior model reflects the balance between the reduced infectiousness of non-symptomatic infectious individuals (exposed and asymptomatic) and the behavioral response of susceptible and non-symptomatic individuals. Our simulations show that risk misperception to silent transmissions increases the attack rate when the epidemic is mainly driven by asymptomatic infections. The lower the risk of infection perceived (via the disease prevalence level), the weaker the behavioral response.

**Figure 3:**
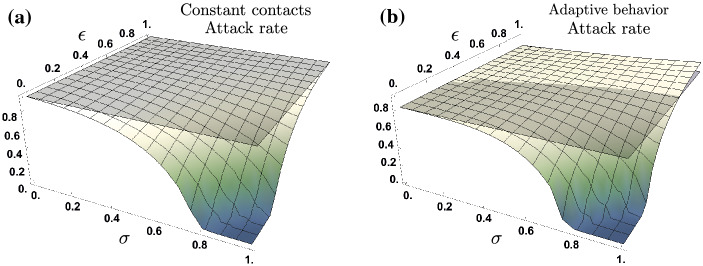
Attack rate under fixed contact rates (**a**), and under adaptive behavior (**b**), as a function of the proportion of asymptomatic infections ($\sigma$) and their relative infectiousness ($\epsilon$). Panel (**a**) shows that under constant contact rates, changes on the proportion of asymptomatic infections and their relative infectiousness monotonically decreases the attack rate. Panel (**b**) shows that under adaptive behavior, there exists scenarios for which the attack rate in the presence of asymptomatic cases overcomes the attack rate of having no asymptomatic cases. For the adaptive behavior model, the presence of asymptomatic cases can increase or decrease the attack rate, relative to their infectiousness. Parameter set $\tau =14,\nu =0.1,C^I=\max C_t$, $l=1$ and $\rho =0.25$.

We found that there is a trade off between the proportion of asymptomatic cases, the reduced infectiousness of asymptomatic individuals and, the behavioral response produced on susceptible and non-symptomatic individuals. In Fig. 3 we explore all the potential scenarios where the attack rate is a function of both the proportion of infections that are asymptomatic ($\sigma$), and their relative infectiousness ($\epsilon$). Panel (a) shows the attack rate for the constant contact rates model, and panel (b) shows the attack rate for the adaptive behavior model. We take the case where there are no asymptomatic infections ($\sigma =0$) as the baseline scenario (gray plane), for each model. Panel (a) shows that under the constant contact rates model, regardless of asymptomatic individuals’ relative infectiousness, an epidemic driven by both symptomatic and asymptomatic cases ($\sigma >0$), leads to a lower attack rate than an epidemic solely driven by symptomatic cases. That is, under the constant contact rates model the attack rate attained for all $(\sigma ,\epsilon )$ scenarios, is lower than the attack rate for the baseline scenario, ($\sigma =0$).

Panel (b) shows as expected, that the attack rate in the absence of asymptomatic infections under behavioral response $(\sigma =0)$, is lower than the corresponding one under constant contact rates. Interestingly, under the adaptive behavior model, the attack rate shows a non-monotonic behavior to the presence of asymptomatic infections. For scenarios where asymptomatic individuals’ infectiousness is high ($\epsilon >0.6$), behavioral response leads to an increased attack rate, relative to the baseline scenario $(\sigma =0)$. In counterpart, for scenarios where asymptomatic individuals’ infectiousness is low ($\epsilon <0.4$), behavioral response leads to a reduced attack rate, compared to the baseline scenario. Notice that for intermediate levels of asymptomatic individuals’ infectiousness, the impact of adaptive behavior on the attack rate depends upon the trade-off between the proportion of asymptomatic cases $(\sigma )$ and their relative infectiousness $(\epsilon )$.

In summary, the set of presented simulations suggest that under adaptive behavior the trade off between the proportion of asymptomatic individuals and their relative infectiousness, defines a threshold. The presence of relatively highly infectious asymptomatic individuals, in conjunction with risk misperception produced by silent transmission, has the potential to generate more cases than the analogous epidemic composed purely by symptomatic transmissions.

### Symptomatic individuals’ behavior modulates the impact of non-symptomatic infections

Our next set of experiments tested the effect of symptomatic individuals’ activity level on the attack rate produced for scenarios varying asymptomatic cases ratio and their relative infectiousness. Specifically, we explored the impact of behavioral responses on the attack rate as the contact rate of infected (but still socially active) individuals varies. We found the attack rate in the presence of asymptomatic cases is modulated by the contact rate of symptomatic but still socially active individuals.

**Figure 4:**
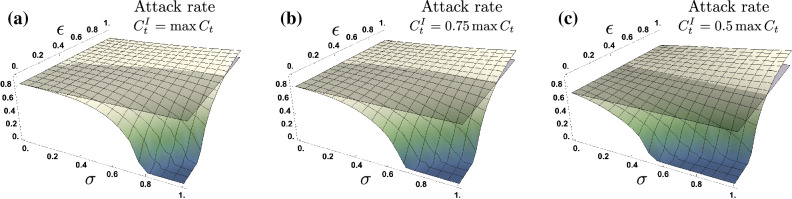
Attack rate as a function of the proportion of asymptomatic infections ($\sigma$) and their relative infectiousness ($\epsilon$), for different contact rates of symptomatic individuals under adaptive behavior model. In panel (**a**) we assume symptomatic individuals maintain the privately optimal contact rate ($C^I_t=\max C_t$), in panel (**b**) we assume symptomatic individuals reduce their contact rate to 75% ($C^I_t=0.75\max C_t$), and in panel (**c**) we assume a contact rate reduction to 50% ($C^I_t=0.5\max C_t$). Compliance with recommended precautionary measures by infectious individuals moderates the impact of non-symptomatic infections. The lower the symptomatic individuals’ contact rate, the greater the impact of non-symptomatic infections on the attack rate. Parameters $\tau =14,\nu =0.1$, and $\rho =0.25$.

Since it is expected that some symptomatic infected individuals comply with health authorities recommendations, we assume that variations in the contact rate of infected individuals are determined by both the ratio of compliant to non-compliant infected individuals (*l*) and the contact rates reduction of compliant individuals ($u_C$). The higher the compliance rate, the lower the contact rate. Figure 4 shows the impact on the attack rate, of the proportion of asymptomatic cases ($\sigma$) and their relative infectiousness ($\epsilon$), for scenarios where symptomatic infected individuals exhibit contact rates of $C^I_t= 100\%, 75\%$ and $50\%$. Our simulations show that, in general, the attack rate of the epidemic decreases as the contact rate of symptomatic individuals falls, an intuitive result. Moreover, Fig. 4 shows two effects on the attack rate as symptomatic contact rates decreases: (i) the impact of non-symptomatic infections increases as the symptomatic individuals are less socially active, increasing the attack rate over the baseline scenario, (ii) the higher the level of compliance (the reduction in the symptomatic individuals’ contact rate), the lower the levels of asymptomatic cases and the relative infectiousness ($\sigma ,\epsilon$) at which the attack rate exceeds the baseline scenario ($\sigma =0$). In other words, the $(\sigma ,\epsilon )$ values that lead to an increased final epidemic size over the base case decrease as symptomatic compliance increases. Furthermore, for the scenarios at which the attack rate in the presence of asymptomatic individuals exceeds the baseline scenario (the no asymptomatic cases scenario), the impact on the attack rate increases as the symptomatic individuals compliance increases.

The intuition behind our result is that by reducing symptomatic individuals activity level, the infection risk perception decreases. However, risk misperception towards non-symptomatic individuals leads silent transmissions to play a preponderant role as mixing occurs mainly between susceptible and non-symptomatic infectious individuals. Moreover, due to the reduced infection risk perception, the mixing between susceptible and non-symptomatic infectious individuals tend to occur at high contact rates, as seen in Fig. 1b.

### Optimal planning horizon minimizing the attack rate

Finally, we considered the impact of the planning horizon of susceptible and non-symptomatic individuals on the attack rate. In the proposed adaptive behavior model the planning horizon is the period over which individuals anticipate the costs and benefits of contact decisions. During the planning horizon individuals assume the disease prevalence to be constant. It may be thought of as the period over which individuals have confidence that the state of the epidemic will remain unchanged. We investigated the sensitivity of the attack rate to variations in the length of the planning horizon as the proportion of asymptomatic infections, and their infectiousness, change.

**Figure 5:**
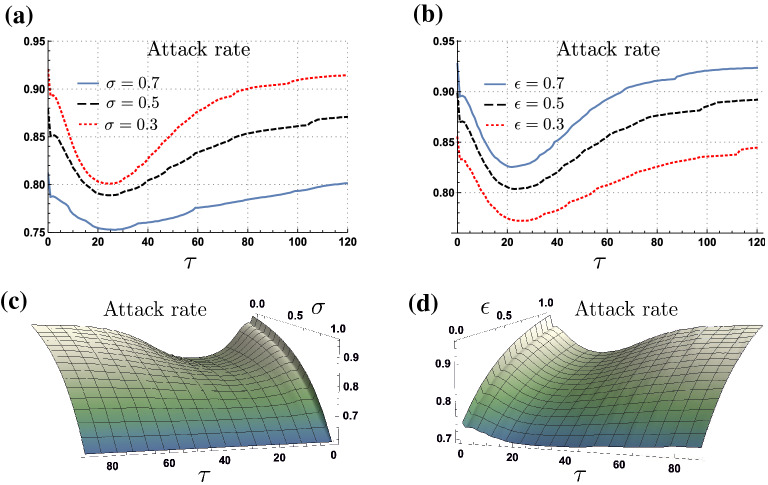
Attack rate as a function of the planning horizon for different proportion of asymptomatic cases and their relative infectiousness, under adaptive behavior model. Panels (**a**) and (**b**) show the non-monotonic effect of increasing the planning horizon on the attack rate, under variations of the proportion of asymptomatic cases and their relative infectiousness, respectively. Panels (**c**) and (**d**) shows the attack rate as a function of the planning horizon and the proportion of asymptomatic cases, and their relative infectiousness, respectively. For $\tau$ between 20 and 25 days the attack rate is minimized for all $\sigma$ and $\epsilon$ scenarios.

We found the attack rate to be sensitive to the length of the planning horizon. Indeed, our simulations suggest there exists a planning horizon that minimizes the impact of the epidemic. Figure 5a shows the attack rate as a function of the length of the planning horizon for the scenarios of 30% ($\sigma =0.3$), 50% ($\sigma =0.5$) and 70% ($\sigma =0.7$) of asymptomatic cases, with relative infectiousness of $\epsilon =0.4$. Figure 5b shows the attack rate as a function of the length of the planning horizon for the scenarios where asymptomatic individuals have a relative infectiousness of 70% ($\epsilon =0.7$), 50% ($\epsilon =0.5$) and 30% ($\epsilon =0.3$), for a proportion of asymptomatic cases of $\sigma =0.5$. Our selected simulations show that the attack rate is minimized for a planning horizon between 20 and 25 days regardless of the proportion of asymptomatic cases and their relative infectiousness.

Figure 5c,d, show the attack rate attained as a function of the planning horizon, for all possible scenarios of asymptomatic ratios and relative infectiousness, respectively. Our numerical experiments show that the existence of the optimal planning horizon is robust to variations on the asymptomatic subpopulation characteristics. That is, the optimal planning horizon is a consequence of the proposed adaptive behavioral response model.

The previous simulations suggest that while the projection of the benefits and costs of making contacts over long planning horizons is beneficial, the assumption of constant prevalence may deviate risk assessments leading to high attack rate values. Moreover, we found the optimal planning horizon to be sensitive to the disease basic reproductive number and, to the expected utility loss related to the infectious compartments $\left( u(I,C^*_t)/u(S,C^*_t)\right)$. Intuitively, the planning horizon length producing the minimal attack rate is the one at which the expected utility appropriately weights the utility loss while infected. Specifically, short planning horizons underweight the expected utility losses of being infected, by potentially missing individuals’ transitions across disease health classes. In counterpart, long planning horizons tend to overweight the expected utility obtained after have gone over the whole disease progression, that is, while recovered.

**Figure 6:**
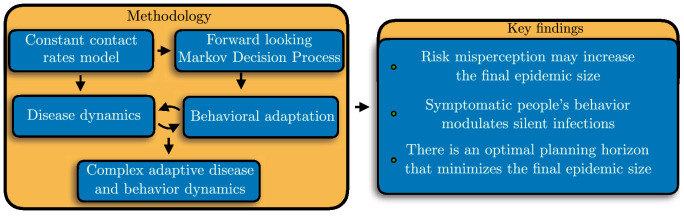
Methodology components of our adaptive behavior model and key results.

Figure 6 summarizes the methodology components of our adaptive behavior model and our key results.

## Discussion

The starting point for this analysis is the finding that adaptive behavior by susceptible and non-symptomatic individuals responding to the perceived infection risk alters epidemic dynamics by dynamically modifying the structure of contacts^40^. In this paper we focused on an important feature of the COVID-19 pandemic: that a large proportion of infected individuals are asymptomatic or have symptoms at a level that allows continued social interaction. Absent testing, infected with mild symptoms and asymptomatic individuals may both behave and be treated by others as if they are susceptible. On the other hand, absent enforcement of health authority recommendations, symptomatic individuals experiencing only mild effects may continue to engage with others.

To uncover the importance of the non-symptomatic proportion of the infected population we considered the impact of behavioral responses to the risks and rewards of contact with others, assuming variable levels of compliance with health authority recommendations on the part of infected individuals. Taking the case where non-symptomatic individuals do not make any attempt to mitigate the risks to themselves or others as the base case, we considered how the inclusion of behavioral responses may be expected to alter disease dynamics. We supposed that individuals do not have perfect knowledge of either their own health class or the health class of others, and that they make decisions based on observable cues—symptoms of disease.

A study using data from New York City, New York and Austin, Texas, found that the attack rate in the first wave of the pandemic had depended on the proportion of asymptomatic infections but not on the infectiousness of asymptomatic individuals^41^. Consistent with this study, we found that while the inclusion of behavioral responses generally reduces the final epidemic size relative to the base case, the effect was highly sensitive to the proportion of the infected population that was asymptomatic. However, we also found the final epidemic size to be highly sensitive to both the infectiousness of the asymptomatic population and to the compliance with health authority recommendations of the symptomatic but socially engaged population. The higher the proportion of the infected population that is asymptomatic, and the greater the infectiousness of asymptomatic individuals, the greater the final epidemic size. Particularly, if there are asymptomatic infections, Fig. 4 shows that there is a threshold determined by the proportion of asymptomatic cases and their relative infectiousness, for which the final epidemic size is larger than would occur if there were no asymptomatic infections. It also shows that the greater the rate of compliance with health authority recommendations by symptomatic individuals, the greater the likelihood that asymptomatic infections will lead to a final epidemic size larger than would occur absent asymptomatic infections.

The evidence to date on both the proportion of infections that are asymptomatic and the relative infectiousness of asymptomatics is mixed. The New York/Austin study reported that 56% of infections were estimated to be asymptomatic^41^. This result is consistent with other studies outside China^42^, but is higher than was found in studies focused on the original outbreak in Wuhan. A study of Japanese evacuees from Wuhan, for example, found the asymptomatic ratio to be 30.8%^12^.

Evidence on the relative infectiousness of symptomatic and asymptomatic individuals indicates that asymptomatic infections may well be increasing the final epidemic size. Most studies have found viral loads in symptomatic and asymptomatic individuals to be similar^43^, but even where viral loads have been found to be lower in asymptomatic individuals, a period of viral shedding has been observed^44^. Modelling exercises have shown that differences in the generation-interval distribution of asymptomatic and symptomatic transmission matter, and can significantly bias estimates of the basic reproduction number^45^. The first quantitative study of asymptomatic transmission found a total infection rate of 6.15%, with 6.30% and 4.11% for symptomatic and asymptomatic individuals respectively^46^. The implication is that the relative infectiousness of asymptomatic individuals is such that the final epidemic size is increasing in the proportion of asymptomatic infections.

Absent large scale random testing there is no way to generate precise estimates of the size of the infected and infectious asymptomatic population, and in consequence no way to generate reliable estimates of the disease reproduction number. However, by investigating changes in observable contact and associated attack rates it may be possible to infer the size and the impact of the infected asymptomatic and pre-symptomatic populations.

The framework we use to model individuals’ adaptive behavior during an epidemic focuses on the private benefits and costs of contacts. The individuals does not consider the impact that their behavior would have on others. The individual does not internalize the external costs and benefits of their behavior. The social costs of private behavior are instead reflected in health authority recommendations on, for example, social distancing measures or the use of personal protective equipment. We explore the consequences of variations in infected individuals’ willingness to comply with such recommendations. Another critical aspect on the model is the uni-dimensional and single peaked utility function. This allows us to focus on the costs and benefits of contact decisions alone, but neglects other factors that may influence individual decisions. The population in health states *S*, *E*, *A*, *R* are assumed to be homogeneous. The population in health state *I* is divided between those who choose to comply with health authority recommendations, and those who do not. They balance the costs and benefits of contact over that horizon assuming no change in prevalence. The benefits of being forward-looking in some state are constrained by the speed at which that state is changing.

While these assumptions allow us to explore the role of human behavioral responses during an epidemic, we recognize that take no account of the many other factors influencing decision-making in the current epidemic. Politicization of the epidemic is partially reflected in the parameter describing compliance with health authority recommendations, but we cannot, for example, capture the very different constraints faced by individuals in manufacturing and services, or the limited capacity to respond by those on low incomes. However, our goal is to capture the interactive evolution of human behavioral adaptation and epidemic dynamics, by using a simple but insightful mechanistic model.

The proposed model assumes susceptible and non-symptomatic (exposed and asymptomatic) individuals are aware of the disease prevalence at each time step, but do not have perfect knowledge of either their own health class or the health class of others. In reality, risk perception depends upon the region-specific level of testing, where the perceived prevalence (the combination of symptomatic and asymptomatic individuals detected) is a fraction of the true epidemic size. In such a scenario, risk misperception not only arises due to asymptomatic individuals but also due to testing limitations. The challenge is exacerbated in regions where testing is very limited and where infectious individuals continue engaging in social interactions. Economic stress and the lack of reasonable alternatives are some of the factors leading the population to risk the dangers of COVID-19^47,48^.

On the other hand, our simulations shown some potential impacts of reducing symptomatic individuals contact rates, for instance due to detection and quarantine or isolation. The modification of the contact structure by reducing symptomatic individuals’ activity has the potential to be balanced, if not overcame, by the increasing contact rates of pre-symptomatic and asymptomatic individuals, producing a comparable or a worse epidemic scenario. Therefore, an effective control measure intended to reduce secondary cases by isolating or quarantining infectious individuals should enforce compliance as well as mass testing, so that an epidemic is not driven by silent transmissions produced due to infection risk misperceptions.

## Methods

### Mathematical model

Our model focuses on infected individuals who are capable of social interaction, i.e., infected individuals who have no symptoms or mild symptoms. Since our goal is to study the impact of the behavior of infectious exposed and asymptomatic individuals on the disease dynamics, we neglect individuals with severe symptoms, since these do not interact with the rest of the population. The potential impact of nosocomial outbreaks has been analyzed in the context of SARS, pneumonia and other diseases^49^.

Our model of disease transmission is composed of susceptible (*S*), pre-symptomatic infectious exposed individuals (*E*), infected individuals with symptoms or testing positive (*I*), infectious but asymptomatic individuals (*A*), and recovered individuals (*R*). We suppose that only individuals in *I* know themselves to be infected either through observation of symptoms or through a positive test result. During the ongoing COVID-19 pandemic, it has been shown that infected individuals carry the highest viral load on or before symptom onset^50^. Due to the lack of adequate data on the specific infectiousness of exposed individuals, we assume this subpopulation to be less infectious than symptomatic individuals, $\rho =0.25$. We explore the impact that changing the exposed individuals’ infectiousness produce on the evolution of the disease transmission and on the attack rate (the proportion of finally infected individuals), in the Supplementary Appendix C section. We assume that on average, $1/\kappa$ days after infection, a proportion $\sigma$ of exposed individuals remain asymptomatic, while the rest develop symptoms.

To capture the fact that only a fraction of the infected population will adopt pro-social precautionary behavior, we stratify the infected population into those who reduce their infectious potential by complying with health authority recommendations ($I_S$), and those who do not ($I_C$)^51^. We assume the fraction *l* of symptomatic individuals do not follow health authority recommendations, while the proportion $1-l$ do it. Individuals may be non-compliant for many different reasons: they may have no reasonable alternative to interact with others, they may be compelled to continue interacting with others, they may be non-compliant for political or ideological reasons, or they may simply be careless. For our purposes all that matters is that a proportion of those known to be infected do not comply with health authority recommendations. The adoption of precautionary measures by the symptomatic population $I_S$ is assumed to reduce their infectious potential by a factor $\eta <1$. All other symptomatic individuals not following precautionary recommendations maintain their infectious potential. Finally, we assume a similar infectious period of $\frac{1}{\gamma }$ days for asymptomatic and symptomatic individuals.

**Figure 7:**
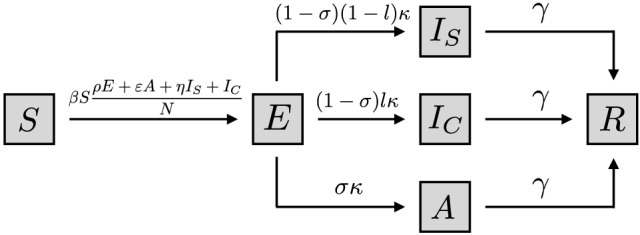
The proposed model for disease progression assumes susceptible (*S*), exposed (*E*), asymptomatic (*A*), symptomatic non-compliant $(I_C)$, symptomatic compliant $(I_S)$, and recovered individuals (*R*).

Our model for disease progression is sketched in Fig. 7 and mathematically described by the system of equations (1).

1 $$\begin{aligned} \begin{aligned} \dot{S}&= -\beta S \frac{\rho E+ \varepsilon A +\eta I_S+ I_C}{N} ,\\ \dot{E}&= \beta S \frac{\rho E+ \varepsilon A +\eta I_S+ I_C}{N} -\kappa E,\\ \dot{I_S}&= (1-\sigma )(1-l)\kappa E-\gamma I_S, \\ \dot{I_C}&= (1-\sigma )l\kappa E -\gamma I_C,\\ \dot{A}&= \sigma \kappa E-\gamma A,\\ \dot{R}&= \gamma (I_S+I_C+ A). \end{aligned} \end{aligned}$$

We computed model’s (1) basic reproductive number,

2 $$\begin{aligned} \mathcal {R}_0=\beta \left( \frac{\rho }{\kappa }+\frac{(1-\sigma )(1-l)\eta }{\gamma }+\frac{(1-\sigma )l}{\gamma }+\frac{\sigma \varepsilon }{\gamma }\right) , \end{aligned}$$

by following the next generation matrix approach, and included the details in Supplementary Appendix A.

### Disease dynamics under adaptive human behavior

Aside from the conditions that lie behind non-compliance with health authority recommendations, we assume a homogeneous population. Changes in health status are the only source of behavioral variation. Individuals’ behavior differs across health classes, but individuals with similar health status behave similarly.

Taking (1) as a baseline, we model the incidence term under adaptive behavior as

3 $$\begin{aligned} \beta C_t^S S \frac{C_t^{E}\rho E+ C_t^{A} \varepsilon A + C_t^{I_S} \eta I_S+ C_t^{I_C} I_C}{C_t^S S+C_t^{E} E+ C_t^{A} A + C_t^{I_S} I_S+ C_t^{I_C} I_C+C_t^{R}R}, \end{aligned}$$

where individuals in health classes $\{S,E,A,I_S,I_C,R\}$ select contact rates $\{C_t^S,C_t^E,C_t^A,C_t^{I_S},C_t^{I_C},C_t^R \}$, at time *t* so as to maximize the discounted stream of net benefits—the present net value—of social interaction. Individuals make contact choices as a function of the health class-specific utility and infection risks of contacts, and this in turn influences the path of the epidemic and hence future contact risks. Observations on current disease prevalence are used to infer infection risks, and hence to project the net benefits of contact over the individual’s planning horizon. Individuals assume the population distribution among health classes and their respective current contact rates are constant over the planning horizon. Risk of infection depends on contact rates $C^h_t$ for $h \in \{S,E,A,I_S,I_C,R\}$, and constitutes a cost that generates a feedback between the epidemiological and economic systems. Formally, individuals in each health class maximize the expected utility of making contacts subject to the dynamics of the epidemic (7).

We determine the contact choices made by individuals at each time step, by finding the contact rate that maximize their expected utility $V_t(h)$ in each of the possible health state $h\in \{S,E,I_S,I_C,A,R\}$, over a given planning horizon, $\tau$. At each time step, the system’s current state (population distribution among health states and their respective contact choices) is assumed to remain constant during the planning period. The expected utility $V_t(h)$ comprises the potential benefit obtained by making the optimal contact choice at each future time step during the planning horizon.

The expected utility comprises the immediate net benefits of contact (which depends only on the individual’s perceived health status), and the expected net benefits of future contacts (which depend on all possible future health states and transitions probabilities). We assume that the utility of making *C* contacts at time *t* is described by a concave single peaked utility function $u_t=u(C_t)$. Individuals obtain positive marginal net benefit from additional contacts up to $C_t^*$, after which additional contacts diminish the net benefits.

Following the work by Morin et al.^29^, we assume a utility function of the particular form $u_t=\left( b C_{t}^{h}-(C_{t}^{h})^ 2\right) ^{\nu }$, where *b* is the maximum number of contacts possible, $\nu$ is the utility function shape parameter, and $C_{t}^{h}$ is the contact rate of a typical individual with health status *h*. Therefore, $u(h,C_t^h)$ is the utility a typical individual in health class *h* obtains by making *C* contacts at time *t*. We assume that individuals get similar per-contact utility regardless of health status, except symptomatic infected individuals who gets no utility during the infectious period. The number of daily contacts maximizing the immediate utility is given by $C^*=b/2$.

To solve the optimization problem, we define a system of Bellman’s equations which are then numerically solved using dynamic programming methods.

#### Non-symptomatic individuals behavior

In the absence of symptoms, we assume exposed and asymptomatic individuals are not aware of their infectious status, perceiving themselves to be susceptible. In consequence, we suppose that non-symptomatic individuals in all three health classes—susceptible, exposed and asymptomatic—choose their contact rates in the same way. All non-symptomatic individuals choose the contact rate that maximizes expected utility over the planning horizon $[t,t+\tau ]$. This is done by weighing current and the expected future benefits of contact against the risk of infection. Expected benefits are conditioned on the probability of future infection, and potential recovery. We model the optimization problem as a dynamic programming problem, the solution to which generates the privately optimal contact rate^27–29^.

Formally, the dynamic programming problem by which susceptible individuals assess the daily optimal contact rate is given by the Bellman’s equation

4 $$\begin{aligned} V_t(S)=\max _{C_t^S}\{u(S,C_t^S)+\delta [(1-P^I)V_{t+1}(S)+P^I V_{t+1}(E) ] \}, \end{aligned}$$

where $V_t(S)$ is the expected utility of susceptible individuals at time *t*, $V_{t+1}(S)$ ($V_{t+1}(E)$) is the expected utility being susceptible (exposed) at time $t+1$, and

5 $$\begin{aligned} P^I=1-\exp \left( -\beta C_t^S S \frac{C_t^{E}\rho E+ C_t^{A} \varepsilon A + C_t^{I_S} \eta I_S+ C_t^{I_C} I_C}{C_t^S S+C_t^{E} E+ C_t^{A} A + C_t^{I_S} I_S+ C_t^{I_C} I_C+C_t^{R}R}\right) \end{aligned}$$

is the probability of being infected at time *t*.

The maximization problem in Eq. (4) accounts for the individual’s immediate utility ($u(S,C_t^S)$), plus the expected future utility discounted at rate $\delta$. The susceptible individual’s expected future utility comprises the expected utility of remaining susceptible with probability $1-P^I$ and, the expected utility of being infected (progressing to the *E* compartment) with probability $P^I$.

Notice that in order to solve Eq. (4), the expected utility of exposed individuals is required, which is given by Eq. (6)

6 $$\begin{aligned} V_t(E)=u(E,C_t^S)+\delta [(1-P^E)V_{t+1}(E)\,+\,P^E(\sigma V_{t+1}(A)+(1-\sigma )[(1-l)V_{t+1}(I_S)+ l V_{t+1}(I_C)]) ], \end{aligned}$$

where $P^E=1-e^{-\kappa }$ stands for the probability of moving from the *E* health class to either *A*, $I_S$ or $I_C$ health classes, with probabilities defined by our constant contact rates model. Similar to Eq. (4), $V_t(E)$ sums the immediate utility of currently being exposed ($u(E,C_t^S)$) and the discounted expected future utility of progressing to possible future health states. The future expected utility while exposed comprises the expected utility of remaining in the exposed compartment with probability $(1-P^E)$ or progressing out of the exposed compartment with probability $P^E$. The future expected utility for exposed individuals progressing to a different health class comprises the future expected utilities of being asymptomatic, infected compliant or infected non-compliant, with probabilities $P^E\sigma , P^E(1-\sigma )(1-l)$ and $P^E(1-\sigma ) l$, respectively.

Finally, the expected utility of asymptomatic ($V_t(A)$), infected compliant ($V_t(I_S)$) and infected non-compliant ($V_t(I_C)$), comprise the immediate utility and the discounted future expected utility when recovered ($V_t(R)$). The Bellman’s equations for individuals in these health states are, respectively:

7 $$\begin{aligned} V_{t}(A)= & {} U(A,C_t^S)+\delta [(1-P^R) V_{t+1}(A)+P^RV_{t+1}(R) ], \end{aligned}$$

8 $$\begin{aligned} V_{t}(I_S)= & {} U(I_S,C_t^{I_S})+\delta [(1-P^R) V_{t+1}(I_S)+P^RV_{t+1}(R) ], \end{aligned}$$

9 $$\begin{aligned} V_{t}(I_C)= & {} U(I_C,C_t^{I_C})+\delta [(1-P^R) V_{t+1}(I_C)+P^RV_{t+1}(R) ], \end{aligned}$$

where $P^R=1-e^{-\gamma }$ is the probability of recovery.

Notice that the assumption that non-symptomatic individuals are unaware of their health status implies that the current utility ($U(h,C_t^S)$ for $h\in \{E,A\}$), is computed choosing a contact rate similar to individuals in the susceptible health state. That is, the contact rates used in the terms $u(E,C_t^S)$ and $u(A,C_t^S)$ in Eqs. (6) and (7), respectively, are driven by individuals’ own health status perception. A variation to the modeling framework proposed in Refs.^27–29^.

#### Symptomatic infected individuals

We suppose that symptomatic infected individuals divide into two sub-classes: a fraction $1-l$ of symptomatic individuals comply with health authority recommendations for the mitigation of population level disease risk ($I_S$), while the rest of symptomatic individuals do not comply with those recommendations ($I_C$). We suppose that all individuals in $I_S$ and $I_C$, develop symptoms and are aware that they are infected and infectious. The solution to the Bellman’s equation for symptomatic infected individuals generates the privately optimal contact rate for individuals in that health class. However, we also suppose that individuals in $I_S$ are willing to reduce their contact rate below the privately optimal level in compliance with health authority recommendations^52^. Particularly, we suppose that compliant infected individuals are willing to accept a reduction in the utility they gain from contacts, so long as utility does not fall below the minimum acceptable level, $u_c$. In this respect, our approach differs from the framework proposed in Refs.^27–29^.

Note that expected utility in (8) and (9) depends on the average recovery period. We therefore derive the following explicit expression for non-compliant $I_C$ individuals’ expected utility

10 $$\begin{aligned} V_t(I_C) = u(I_C,C^{I_C*}) \displaystyle \sum _{j=1}^\tau \delta ^j (1-P^R)^j + u(R,C^{R*}) \displaystyle \sum _{j=1}^\tau \delta ^j\left( 1-(1-P^R)^j \right) , \end{aligned}$$

where $C_t^{I_C*}\le C_t^*$. The first term of (10) corresponds to the expected utility obtained while infected, and the second term corresponds to the expected utility obtained while recovered, during the planning horizon.

By contrast, compliant individuals reduce their contact rate subject to a level consistent with securing a minimal utility, solving the problem

11 $$\begin{aligned} V_t(I_S) =\min _{C_t^{I_S}} \lbrace u(I_S,C_t^{I_S}) \displaystyle \sum _{j=1}^\tau \left( \delta (1-P^R) \right) ^j\rbrace ,  \text {subject to}  \frac{V_t(I_S)}{\tau }>u_C. \end{aligned}$$

The critical utility value for compliant individuals is a free parameter that allow us to calibrate the model for different scenarios of compliance. Notice that Eq. (11) comprises only the infectious period, since an infected individual is assumed to stop this behavioral regime when recovered.

Taking into account the contact rates of compliant and non-compliant individuals, we let the expected contact rate of symptomatic individuals to be given by weighting the non-compliant and compliant individuals with their respective contact rates: $C^I_t=l(C^{I_C})+(1-l)C^{I_S}_t$.

#### Recovered individuals

We assume there is no incentive for recovered individuals to behave strategically, since our model does not consider potential reinfections. Therefore, we let recovered individuals make the daily number of contacts that maximizes the net benefits of contact. The recovered individuals Bellman’s equation is given by

12 $$\begin{aligned} V_{t}(R)=u(R,C_t^{R*})+\delta V_{t+1}(R). \end{aligned}$$

## Supplementary Information

Supplementary Information.
