# Supplementary material for: Asymptomatic individuals can increase the final epidemic size under adaptive human behavior
Source: Sci Rep. 2021 Oct 5;11:19744. doi: 10.1038/s41598-021-98999-2 (PMC8492713; doi:10.1038/s41598-021-98999-2)
Supplement: Supplementary file 1 — Supplementary Information. [file 41598_2021_98999_MOESM1_ESM.pdf]

Supplementary Material
Asymptomatic individuals can increase the final epidemic size under adaptive human behavior

Baltazar Espinoza[1], Madhav Marathe[1] Samarth Swarup[1], Mugdha Thakur[1]

1. Biocomplexity Institute and Initiative, Network Systems Science and Advanced Computing Division, University of Virginia, Virginia, USA

A Constant contacts model's basic reproductive number and final epidemic size

We use the next generation approach [1, 2], to compute Model's (1) basic reproductive number. We consider the infectious compartments E, I and A and define

$$\mathcal{F} = \begin{pmatrix} \beta S \frac{\rho E + \varepsilon A + \eta I_S + I_C}{N} \\ 0 \\ 0 \\ 0 \end{pmatrix} \quad \text{and} \quad \mathcal{V} = \begin{pmatrix} -\kappa E \\ \gamma I_S - (1-\sigma)(1-l)\kappa E \\ \gamma I_C - (1-\sigma) l \kappa E \\ \gamma A - \sigma \kappa E \end{pmatrix},$$

where the next generation matrix is given by $-FV^{-1}|_{S=N}$ and the basic reproductive number given by its spectral radius

$$\mathcal{R}_0 = \beta \left(\frac{\rho}{\kappa} + \frac{(1-\sigma)(1-l)\eta}{\gamma} + \frac{(1-\sigma)l}{\gamma} + \frac{\sigma\varepsilon}{\gamma} \right), \tag{A.1}$$

with F and V representing the Jacobian matrices of $\mathcal{F}$ and ν, respectively. The basic reproductive number (A.1) accounts for the average secondary infections produced by exposed $\left(\beta \frac{\rho}{\kappa}\right)$, symptomatic compliant $\left(\beta \frac{(1-\sigma)(1-l)\eta}{\gamma}\right)$, symptomatic non-compliant $\left(\beta \frac{(1-\sigma)l}{\gamma}\right)$ and asymptomatic individuals $\left(\beta \frac{\sigma\varepsilon}{\gamma}\right)$. Notice that in the absence of compliant individuals ($l = 1$), asymptomatic infections ($\sigma = 0$), and infectious exposed individuals ($\rho = 0$), the basic reproductive number takes the traditional form $\mathcal{R}_0 = \frac{\beta}{\gamma}$.

During the initial stage of an epidemic, the disease initially propagates in the absence of sanitary recommendations on a population almost completely susceptible. In the absence of human adaptive response, the potential of an epidemic to propagate among the population is captured by its basic reproductive number. We use expression (A.1) to explore the trade-off between the proportion of initially asymptomatic infections and the proportion of symptomatic individuals that makes an epidemic mathematically sustainable ($\mathcal{R}_0 > 1$), in the absence of behavioral response.

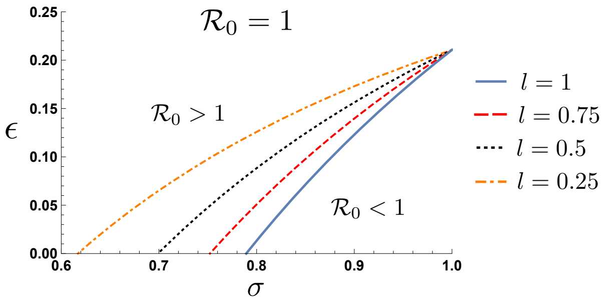

Figure A.1: The trade-off between the proportion of asymptomatic and their relative infectiousness producing $\mathcal{R}_0 = 1$, for scenarios where of 25%, 50%, 75% and 100% of the symptomatic individuals are non-compliant ($l = 0.25, 0.5, 0.75$ and $l = 1$, respectively). A COVID-19 like epidemic, initially propagating in the absence of sanitary recommendations ($l = 1$ and $\mathcal{R}_0 = 2.4$), is mathematically sustainable whenever the proportion of asymptomatic cases is less than 80%, regardless of their relative infectiousness. Parameter values: $\gamma = \frac{1}{9}, \kappa = \frac{1}{5}, \beta = 0.01324, C_t = 48, \rho = 0.25$ and $\eta = 0.4$.

Figure A.1 shows the level curves $\mathcal{R}_0 = 1$ as a function of the proportion of asymptomatic individuals (σ) and their relative infectiousness (ϵ), for scenarios where of 25%, 50%, 75% and 100% of the symptomatic individuals are non-compliant (l). Our simulations show that a COVID-19 like epidemic, initially propagating in the absence of sanitary recommendations ($l = 1$ and $\mathcal{R}_0 = 2.4$), is mathematically sustainable whenever the proportion of asymptomatic cases is less than 80%, regardless of their relative infectiousness. In other words, at the early stage of the epidemic, a force of infection driven solely by symptomatic cases is enough to propagate the disease among the population.

We derive the final epidemic size relation and the attack rate relation of the constant contact rates model, as function of the proportion of asymptomatic cases and their relative infectiousness. By assuming $S(0) = N$, $E(0) = I_S(0) = I_C(0) = A(0) = R(0) = 0$ and, by adding the first two equations in model (1) in the main text we get $\dot{S} + \dot{E} = -\kappa E$, then $E^\infty = 0$ and $\hat{E} = (N - S^\infty)\frac{1}{\kappa}$, where $\hat{f}(t) = \int_0^\infty f(s)ds$ and $f^\infty = \lim_{t\to\infty} f(t)$. Following the same reasoning $\hat{I}_S = (N - S^\infty)\frac{(1-\sigma)(1-l)}{\gamma}$, $\hat{I}_C = (N - S^\infty)\frac{(1-\sigma)l}{\gamma}$ and, $\hat{A} = (N - S^\infty)\frac{\sigma}{\gamma}$. From model's (1) first equation the secondary infections in the population are given by the final epidemic size relation

$$\begin{aligned}\log\left(\frac{N}{S^\infty}\right) &= \beta\left(\frac{\rho}{\kappa} + \frac{(1-\sigma)(1-l)\eta}{\gamma} + \frac{(1-\sigma)l}{\gamma} + \frac{\sigma\varepsilon}{\gamma}\right)\left(1 - \frac{S^\infty}{N}\right)\\ \log\left(\frac{N}{S^\infty}\right) &= \mathcal{R}_0\left(1 - \frac{S^\infty}{N}\right).\end{aligned} \tag{A.2}$$

Denoting the proportion of susceptible individuals at the end of the epidemic by $s^\infty = \frac{S^\infty}{N}$, it follows that $y = 1 - s^\infty$ denotes the proportion of infected individuals during the course of the epidemic, the attack rate. By using expression (A.2) we get

$$\log(s^\infty) = (s^\infty - 1)\mathcal{R}_0 \tag{A.3}$$

which in terms of infected indidivuals becomes

$$y = 1 - \exp(-y\mathcal{R}_0) \tag{A.4}$$

where the solution $y*$ of (A.4), stands for the attack rate and, where $\mathcal{R}_0$ is a function of the proportion of asymptomatic cases and their relative infectiousness $\mathcal{R}_0(\epsilon, \sigma, \rho, \eta, l)$.

B Optimal behavioral decisions via Bellman's equations and dynamic programming

In this section we illustrate the dynamic programming method we use to solve the proposed Bellman's equations via backward induction over the planning horizon $[t, t+\tau]$. In order to obtain the optimal contact rate at current time t, we make use of the *Bellman's principle of optimality.* The dynamic optimization problem over the whole planning horizon is split into a sequence of subproblems over sequencial periods $[t, t+1]$, $[t+1, t+2]$,$\cdots$,$[t+\tau-1, t+\tau]$.

To solve the dynamic programming problem backwards on time we need boundary conditions. Notice that the following relations holds regardless of the individual's health status h,

- $V_{t+\tau+1}(h) = 0$, since it exceeds the boundary of the planning horizon $[t, t+\tau]$,
- $V_{t+\tau}(h) = u_{t+\tau}(h)$, since it is the last period of the planning horizon.

Now, let's analyze backwards induction method to solve susceptible individuals' Bellman's equation. To find the optimal contact rate at current time, we assume a constant projection of the system's current state.

- **Susceptible individual's optimal contact choice at time** $t+\tau$
  Since this is the last period of the planning horizon, individuals do not assess future risk or benefits beyond the current day. Therefore the optimal decision at $t+\tau$ is the contact rate that maximizes individuals' immediate utility,
  $$V_{t+\tau}(S) = u(S, C^*), \quad \text{and} \quad V_{t+\tau}(E) = u(E, C^*)$$
  where $C^* = b/2$.

- **Susceptible individual's optimal contact choice at time** $t+\tau-1$
  To find the optimal contact rate at this period we should make use of the previously found optimal choice at time $t+\tau$. The optimization problem becomes
  $$\begin{aligned} V_{t+\tau-1}(S) &= \max_{C^S_{t+\tau-1}} \{u(S, C^S_{t+\tau-1}) + \delta[(1-P^I)V_{t+\tau}(S) + P^I(V_{t+\tau}(E))]\}, \\ &= \max_{C^S_{t+\tau-1}} \{u(S, C^S_{t+\tau-1}) + \delta[(1-P^I)u(S, C^*) + P^I(u(E, C^*))]\}, \end{aligned}$$
  finally
  $$V_{t+\tau-1}(S) = \max_{C^S_{t+\tau-1}} \{u(S, C^S_{t+\tau-1}) + \delta[(1-P^I)u(S, C^*) + P^I(u(E, C^*))]\} \tag{B.1}$$
  where P^I (the probability of being infected) is known from the system's state at time t
  $$P^I = 1 - \exp\left(-\beta C^S_t S \frac{C^E_t \rho E + C^A_t \varepsilon A + C^{I_S}_t \eta I_S + C^{I_C}_t I_C}{C^S_t S + C^E_t E + C^A_t A + C^{I_S}_t I_S + C^{I_C}_t I_C + C^R_t R}\right).$$
  The optimal contact rate for the period $t+\tau-1$, can be obtained from equation (B.1) by evaluating all the possible contact rates.

- **Susceptible individual's optimal contact choice at time** $t+\tau-2$
  To find the optimal contact rate at this period we should make use of the optimal choices at $t+\tau$ and $t+\tau-1$. The problem becomes
  $$V_{t+\tau-2}(S) = \max_{C^S_{t+\tau-2}} \{u(S, C^S_{t+\tau-2}) + \delta[(1-P^I)V_{t+\tau-1}(S) + P^I(V_{t+\tau-1}(E))]\}, \tag{B.2}$$

notice that we require $V_{t+\tau-1}(E)$ to solve equation (B.2). Here we make use of the Bellman's equation for exposed individuals, at the period $t+\tau-1$,

$$\begin{aligned} V_{t+\tau-1}(E) =& u(E, C^S_{t+\tau-1}) + \delta[(1-P^E)V_{t+\tau}(E) \\ &+ P^E(\sigma V_{t+\tau}(A) + (1-\sigma)[(1-l)V_{t+\tau}(I_S) + lV_{t+\tau}(I_C)])], \\ =& u(E, C^S_{t+\tau-1}) + \delta[(1-P^E)u(E, C^*) \\ &+ P^E(\sigma u(A, C^*) + (1-\sigma)[(1-l)u(I_S, C^*) + lu(I_C, C^*)])], \end{aligned}$$

that is

$$\begin{aligned} V_{t+\tau-1}(E) =& u(E, C^S_{t+\tau-1}) + \delta[(1-P^E)u(E, C^*) \\ &+ P^E(\sigma u(A, C^*) + (1-\sigma)[(1-l)u(I_S, C^*) + lu(I_C, C^*)])]. \end{aligned} \tag{B.3}$$

Notice that we use $u(E, C^S_{t+\tau-1})$ instead of $u(E, C^E_{t+\tau-1})$ since we assume exposed individuals are unaware of their health status and chose the contact rate that corresponds to the susceptible health state. Therefore, $V_{t+\tau-1}(E)$ is known, and equation (B.2) can also be solved by optimizing over all the possible contact rates.

By continuing the backward induction it is possible to solve for the optimal contact rate at each time during the planning horizon, and particularly for the current time t. Since in our epidemic model we only care about the optimal contact rate for the period $t+1$, we use C^S_{t+1} as the contact rate chosen by susceptible and non-symptomatic individuals to run the epidemic model one period ahead.

Table B.1: Recursive dependence of the optimization problem

Health class	$t+\tau-3$	$t+\tau-2$	$t+\tau-1$	$t+\tau$
S	S, E, I_S, I_C, A, R	S, E, I_S, I_C, A	S, E	S
E	E, I_S, I_C, A, R	E, I_S, I_C, A, R	E, I_S, I_C, A	E
I_S	I_S, R	I_S, R	I_S, R	I_S
I_C	I_C, R	I_C, R	I_C, R	I_C
A	A, R	A, R	A, R	A
R	R	R	R	R

C Sensitivity analysis

Summary

Table C.1: Sensitivity summary

Parameter	Description	Range
ρ	Exposed ind. infectiousness	[0, 0.5]
b	Maximum number of contacts per day	[24, 48]
ν	Utility function shape parameter	[0.05, 0.2]
δ	Discount factor	[0.99939, 0.99986]

- Increments in the exposed individuals infectiousness ρ
  - Increases the peak size and the attack rate.
  - Reduces the peak time.
  - Produce stronger adaptive response: increases contacts reduction during the peak time.
  - Reduces the asymptomatic infectiousness ϵ for which the attack rate overcome the attack rate in the absence of asympto
- Maximum number of daily contacts b
  - We found the adaptive behavior model to be low sensitive to changes in b.
- Increments in the utility function shape parameter ν
  - Increase immediate utility and the marginal benefit of increasing contacts.
  - Delay the adaptive response and, in consequence the peak time and the attack rate.
  - Produce a weaker adaptive response: lower reduction of contacts.
- Increments in the annual discount rate δ
  - We found the adaptive behavior model to be low sensitive to changes in δ.

Exposed individuals infectiousness ρ

Since the infectiousness of infectious pre-symptomatic individuals is unknown, for our simulations we assumed the exposed sub-population have a reduced infectious rate relative to symptomatic individuals, $\rho = 0.25$. In this section we aim to study the impact of variations in the relative infectiousness of exposed individuals on the evolution of disease transmission and the final epidemic size.

Selected simulations in Figure C.1 show the impact of the relative infectiousness of exposed individuals on the evolution of the disease dynamics for the constant contacts model (dashed lines) and the adaptive behavior model (solid lines). We explored the scenarios where exposed individuals are 0%, 25% and 50% as infectious as symptomatic individuals, for the set of parameters in Table (1) with $\sigma = 0.3$ and $\epsilon = 0.6$.

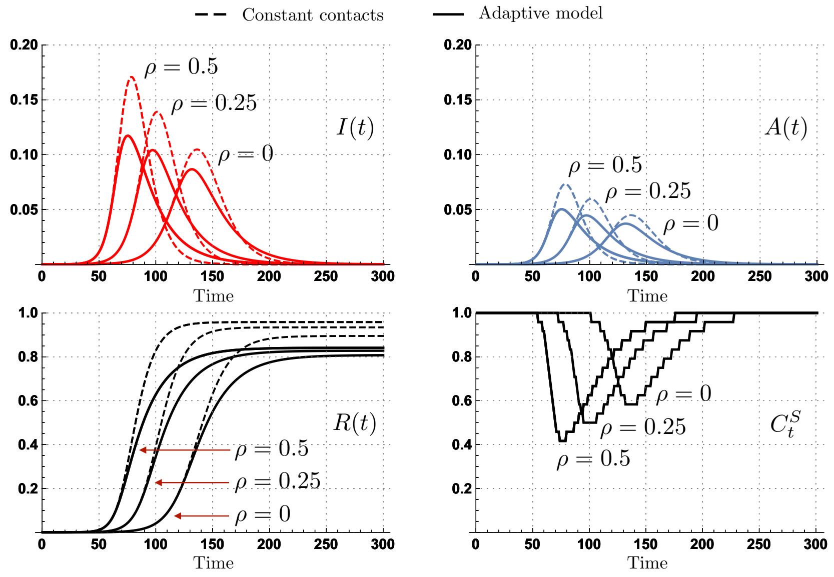

Figure C.1: Disease dynamics under constant contact rates (dashed lines) and under adaptive behavior (solid lines). Exposed individuals' relative infectiousness of $\rho = 0, 0.25$ and 0.5, for the parameters set in Table (1) with $\sigma = 0.3$ and $\epsilon = 0.6$. Increments on the exposed individuals' relative infectiousness increases the peak size and decreases the peak time. Moreover, the final epidemic size is increases and the behavioral response.

Greater exposed individuals' relative infectiousness increases the peak size and the final epidemic size, while decreasing the peak time. In these scenarios, the adaptive response is triggered earlier but at the same prevalence level. Moreover, higher ρ values lead to greater reduction of contacts.

In Figure C.2 we explore the impact of changes in the relative infectiousness of exposed individuals on the attack rate, for the cases where $\rho = 0, 0.25$ and 0.5. We focus on the (σ, ϵ) scenarios producing an attack rate greater than the one corresponding to the baseline scenario, no asymptomatic infections ($\sigma = 0$).

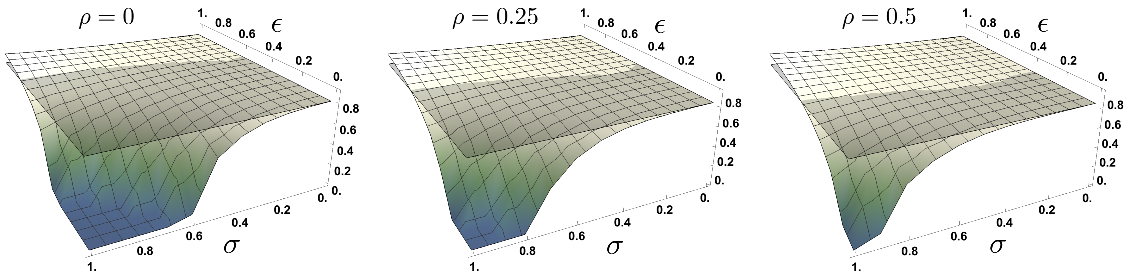

Figure C.2: Attack rate as a function of the proportion of asymptomatic infections (σ) and their relative infectiousness (ϵ), for exposed individuals infectiousness of $\rho = 0, 0.25$ and 0.5. The greater the exposed individuals' infectiousness the lower the asymptomatic individuals' infectiousness required to have an attack rate over the baseline scenario.

Figure C.2 shows that the greater the infectiousness of exposed individuals, the greater the attack rate for all (σ, ϵ) scenarios. Moreover, greater ρ values reduce the asymptomatic individuals' infectiousness (ϵ) required to produce an attack rate above the baseline scenario (gray plane). In other words, the impact of risk misperception increases as the infectiousness of non-symptomatic individuals increases. In addition, high infectiousness of exposed individuals increase the (σ, ϵ) scenarios for which an outbreak is produced.

The utility function $u(C) = \left(bC - C^2\right)^{\nu}$

Due to the absence of appropriate data to calibrate our behavior model, specifically the parameters used in the utility function, in this section we test the sensitivity of the behavioral response and disease dynamics to

changes in the utility parameters. We found the disease dynamics obtained with the behavior model to be low sensitive to the assumed amount of available contacts per day, (b). For an assumed b value we adjust the per-capita likelihood of infection β, so that the basic reproductive number of the behavior model (C.1), matches the targeted basic reproductive value of 2.4,

$$\mathcal{R}_0(C) = C^*\beta\left(\frac{\rho}{\kappa} + \frac{(1-\sigma)(1-l)\eta}{\gamma} + \frac{(1-\sigma)l}{\gamma} + \frac{\sigma\varepsilon}{\gamma}\right), \tag{C.1}$$

where C^* corresponds to the optimal contact rate in the absence of disease.

Figure C.3 shows the disease dynamics for the constant contact rate (dashed lines) and for the behavior model (solid lines), for the scenarios where the maximum daily contact rate of 48 and a per-contact likelihood of infection $\beta = 0.01324$ (panel a), maximum daily contact rate of 24 and a per-contact likelihood of infection $\beta = 0.02649$ (panel b). We found the disease dynamics and the behavioral response produced in both scenarios to be equivalent. Both scenarios produce an attack rate of about 80% and the behavioral response reduce the amount of contacts to 50% during the peak of the epidemic. That is, the higher the amount of contacts assumed, the higher the resolution of the behavioral change over time obtained.

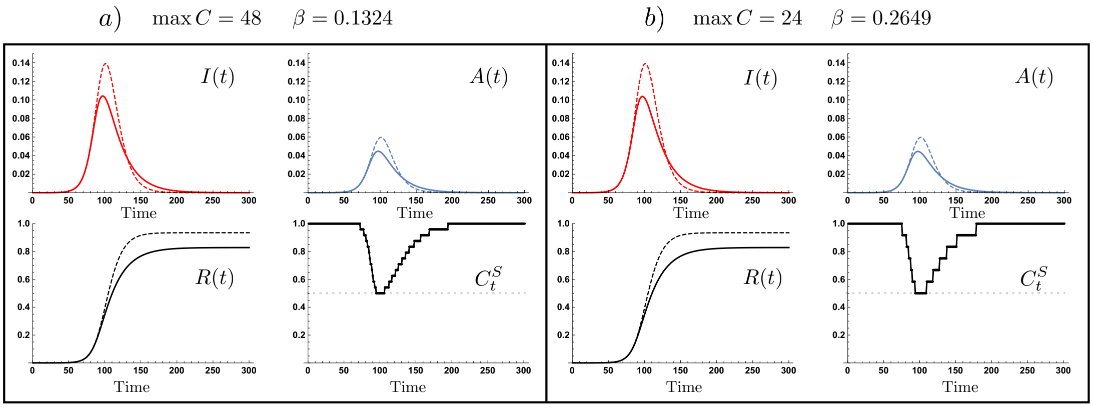

Figure C.3: Disease dynamics under adaptive behavior for different contact rates: in panel a) the maximum contact rate of $b = 48$ leads to $\beta = 0.01324$, in panel b) the maximum contact rate $b = 24$ leads to $\beta = 0.02649$. The normalized contact rate curves in both scenarios $\left(C_t/C^*\right)$ is taken down to a 50% reduction of contacts during the peak of the outbreak. In both scenarios, parameter sets produce a basic reproductive value is $\mathcal{R}_0 = 2.4$. In both scenarios the adjusted basic reproductive values is $\mathcal{R}_0 = 2.4$. For parameters in Table (1) with $\sigma = 0.3$ and $\epsilon = 0.6$

Utility function shape parameter ν

In the proposed behavior model, adaptive response is triggered by assessing the benefits of making contacts while being exposed to the risk of infection. Due to the lack of appropriate data to calibrate the utility function, for our numerical experiments we arbitrarily chose $\nu = 0.1$. Here, we explore the impact that variations in the utility function shape parameter (ν) produce on the disease dynamics.

Figure C.4 panel a) shows the immediate utility obtained by individuals making C contacts per day for ν values of 0.05, 0.1 and 0.2, and where the maximum number of contacts per day is $b = 48$. Lower ν values, makes contacts to be less valuable by producing a lower utility ($u(C,\nu_1) > u(C,\nu_2)$ for $\nu_1 > \nu_2$). We found the behavior model to be highly sensitive to this parameter since the risk-benefit trade-off is directly impacted by the utility obtained by making contacts. Specifically, reducing the amount of utility obtained results in an earlier start of behavioral response. Moreover, Figure C.4 panel b) shows that low ν values reduces the marginal benefit of increasing contacts. Producing an stronger adaptive response since for lower ν values it is “cheaper” to modify contact rates, as compared to bigger ν values.

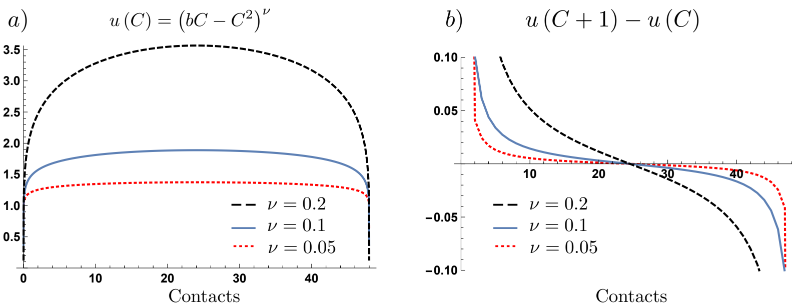

Figure C.4: Figure shows the immediate utility obtained by individuals making C contacts (panel a) and the marginal benefit of increasing contacts (panel b), for ν values of 0.05, 0.1 and 0.2, and maximum daily contacts of $b = 48$. The immediate utility obtained by making C contacts and the marginal benefit of increasing contacts decrease as the ν value decreases.

Figure C.5 show the disease dynamics and adaptive response obtained for the constant contact rates model (dashed lines), and for the adaptive behavior model, using the utility shape parameter values $\nu = 0.05, 0.1$ and 0.2, (solid, dotted, and dot-dashed lines respectively).

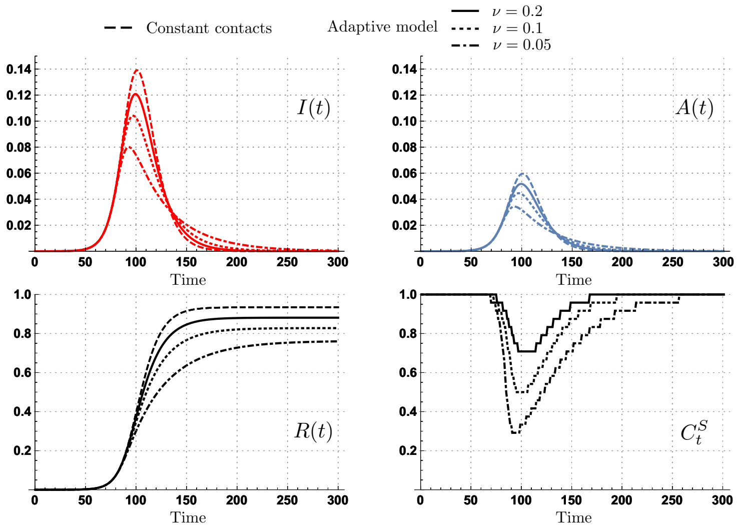

Figure C.5: Disease dynamics under constant contact rates (dashed lines) and under adaptive behavior, for utility shape parameter $\nu = 0.05, 0.1$ and 0.2 (solid, dotted and dot-dashed lines respectively), for parameters in Table (1) with $\sigma = 0.3$ and $\epsilon = 0.6$. Reductions in the utility shape function parameter (ν), produces an earlier start of behavioral adaptation as well as a greater reduction in the contacts during the outbreak.

Reductions on the utility shape function parameter (ν), highly impact the disease dynamics and the adaptive response produced. Decreasing the ν value reduces the amount of utility obtained per contact as shown in Figure C.4 panel a). In consequence starting the adaptive response earlier and producing a greater contacts curtail. This impacts the disease dynamics in several ways, the peak size and the final epidemic size substantially decrease. However, the epidemic period is increased and the peak time is reduced.

The future utility discount rate δ

Our model of behavioral adaptation assumes individuals base their decisions in a projection of the future system's state over a finite planning horizon. Our simulations assume that individuals' future utility has a 5% annual discount rate. However, this parameter may be highly uncertain across individuals, for instance, due to economic or local sanitary conditions.

In this section we study the joint impact of the discount rate and the planning horizon on the optimal behavioral response produced at different instances of the epidemic. We show the optimal decisions that

susceptible and non-symptomatic individuals would make at three instances of the epidemic period, as a function of the discount rate and the planning horizon.

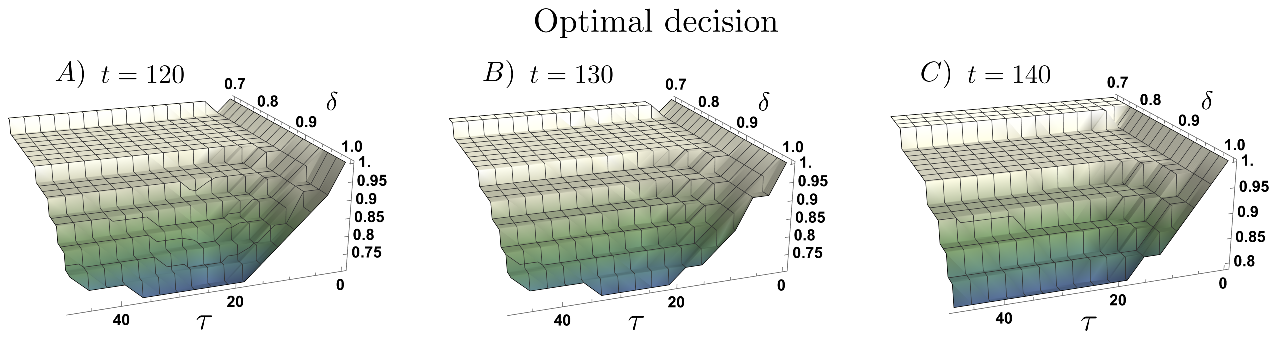

Figure C.6: Susceptible and non-symptomatic individuals' optimal behavioral decisions as a function of the discount rate δ in $[0.7, 1]$ and planning horizons τ in $[0, 40]$, for epidemic instances $t = 120, 130$ and $t = 140$. The bigger the discount rate (δ) the less sensitive the system is to the planning horizon. Different instances of the epidemic drive differential behavioral responses among the susceptible and non-symptomatic individuals.

Figure C.6 panels (A-C) show the optimal decisions made at times $t = 120, 130$ and $t = 140$, for discount rates δ in $[0.7, 1]$ and planning horizons τ in $[0, 50]$. We chose the set of parameters in Table (1) to obtain the state of the epidemic at the selected instances.

Our simulations show that for a given planning horizon, relatively low discount rates ($\delta \approx 1$) lead to strong behavioral responses. Where the strength of the behavioral response also depends on the system's state. In general, we found the optimal decision to be more sensitive to changes in the planning horizon when the discount rate is low.

References

[1] O. Diekmann, J. A. P. Heesterbeek, and J. A. J. Metz. On the definition and the computation of the basic reproduction ratio R_0 in models for infectious diseases in heterogeneous populations. *J. Math. Biol.*, 28(4):365–382, 1990.

[2] P. van den Driessche and J. Watmough. Reproduction numbers and sub-threshold endemic equilibria for compartmental models of disease transmission. *Math. Biosci.*, 180:29–48, 2002.
